# The impact of the 2019 ESC/EAS dyslipidaemia guidelines on real-world initial lipid-lowering therapy in patients with acute myocardial infarction

**DOI:** 10.1097/MD.0000000000037637

**Published:** 2024-03-22

**Authors:** Xiangqi Kong, Gang He, Xiaoqing Quan, Zhixiong Tan, Fengjuan Yan, Xiehui Chen

**Affiliations:** a Shenzhen Longhua District Central Hospital; b Department of Traditional Chinese Medicine, Shenzhen People’s Hospital (The First Affiliated Hospital, Southern University of Science and Technology).

**Keywords:** ezetimibe, lipid-lowering therapy, PCSK9 inhibitors, real-world study

## Abstract

This study aimed to investigate the impact of the latest guidelines on the real-world clinical practice of initial lipid-lowering therapy, especially on the use of ezetimibe and proprotein convertase subtilisin/kexin type 9 (PCSK9) inhibitors in China. All adult patients diagnosed with acute myocardial infarction in our hospital between August 31, 2018, and August 31, 2020, were divided into the following 2 groups: those patients treated before the latest guideline release, and those patients treated after the release. A propensity score-matched method was used, and logistic regression was used to assess the association with intensive statin, ezetimibe and PCSK9 inhibitor usage together with treatment results between the 2 groups. A total of 325 patients were enrolled in this study, including 141 patients who were admitted before the release of the latest guideline and 184 patients who were admitted after the release. After a median follow-up time of 8.20 months, the mean low-density lipoprotein cholesterol was 1.87 ± 0.59 mmol/L (1.87 ± 0.55 in the before group vs 1.88 ± 0.62 in the after group, *P* = .829). After propensity score matching, the initial usage of intensive statin therapy was decreased after guideline release without statistical significance (17.00% vs 28.00%, *P* = .090), whereas the usage of ezetimibe and PCSK9 inhibitors was increased (19.00% vs 8.00%, *P* = .039; and 10.00% vs 3.00%, *P* = .085, respectively). In logistic regression models, the release of the guideline was associated with a statistically significantly increased use of ezetimibe (odds ratio [OR]: 1.91; 95% confidence interval [CI]: 1.21, 3.02; *P* = .005), a marginally decreased use of intensive statins (OR: 0.68; 95% CI: 0.45, 1.03; *P* = .069) and a marginally increased use of PCSK9 inhibitors (OR: 1.31; 95% CI: 0.98, 1.76; *P* = .068). In this single-center, real-world data analysis, after the release of the 2019 European Society of Cardiology/European Atherosclerosis Society guidelines, an increasing number of patients with a recent acute myocardial infarction were initially receiving ezetimibe and PCSK9 inhibitors.

## 1. Introduction

On August 31, 2019, the updated guideline for the management of dyslipidaemias was released by the European Society of Cardiology (ESC)/European Atherosclerosis Society (EAS).^[1]^ For patients with acute myocardial infarction (AMI), the guidelines now recommend the achievement of a low-density lipoprotein cholesterol (LDL-C) reduction of ≥50% from baseline and an LDL-C level of <1.4 mmol/L (<55 mg/dL) (Class 1, Level A). To reach the LDL-C target, the use of add-on therapy with ezetimibe (Class 1, Level B) and a proprotein convertase subtilisin/kexin type 9 (PCSK9) inhibitor (Class 1, level A) is recommended. The new guidelines present a much more aggressive LDL-C target for patients with MI compared with the previous 2016 ESC/EAS guidelines, which recommended an LDL-C goal of <1.8 mmol/L and an LDL-C reduction of ≥50% if the baseline level was 1.8 to 3.5 mmol/L.^[2]^ At present, almost 2 years after the release of the guideline, the impact of the latest guideline on real-world practice and the implications of the new guidelines in terms of the proportion of patients who will be eligible for treatment with ezetimibe and PCSK9 inhibitors are unknown. Furthermore, the initial lipid-lowering therapy before discharge was crucial for patients suffering an MI based on several real-world studies.^[3–5]^ Therefore, this study aimed to investigate the impact of the latest guidelines on real-world clinical practice, especially on the use of ezetimibe and PCSK9 inhibitors in China.

## 2. Material and methods

### 2.1. Study population

We included all adult patients diagnosed with AMI in our hospital between August 31, 2018, and August 31, 2020, who had survived until discharge and who had available follow-up data. For patients with more than 1 admission during the study period, 1 random admission was selected. The following patients were excluded: without data on statin or ezetimibe therapy at admission for the index MI, without LDL-C data at the index MI, died in 3 months after discharge, and without any follow-up visits, including missing LDL-C data during follow-up. All of the information was stored in a cardiovascular disease repository that was integrated with the electronic health record system. The study was approved by the Ethics Committee of Fuwai Hospital Chinese Academy of Medical Sciences, Shenzhen, and no informed consent was needed.

### 2.2. Data variables

To assess the target of a ≥50% reduction in LDL-C level, a baseline LDL-C level is needed. Both baseline and last LDL-C results during follow-up were measured with standard methods in the laboratory in our hospital. Baseline LDL-C was defined as the first result during the index MI hospitalization. The last LDL-C was defined as the most recent result during follow-up. Data regarding initial lipid-lowering therapy medications, including statins, ezetimibe and PCSK9 inhibitors, as well as doses at discharge, were retrieved from the electronic prescribed system and double confirmed by the investigators. An intensive statin was defined as atorvastatin ≥40 mg QD, rosuvastatin ≥20 mg QD or pitavastatin ≥4 mg QD, according to previous studies.^[6]^

The index MI was categorized as a non-ST segment elevation myocardial infarction and ST segment elevation myocardial infarction based on clinical diagnoses at the time of discharge. Past medical history was retrieved from clinical notes by the investigators, and all of the definitions followed the specific guidelines and were predefined as structured input integrated into the electronic health record system, which ensured that there were no missing values. Vital signs, including systolic blood pressure, diastolic blood pressure, pulse, and body mass index, were collected from structured electronic physical examination notes.

### 2.3. Statistical analyses

In general, continuous variables are described as the mean ± SD, and categorical variables are presented as the number (percentage). Differences in the various characteristics among the groups were compared by using the *t* test and χ^2^ test.

Although statins and ezetimibe are well-established treatments and are available at a relatively low cost, PCSK9 inhibitors have a higher cost, and some physicians may not consider these drugs for patients who are close to their LDL-C target. Three types of patients who attained the LDL-C target were defined in our study: patients who had reached an LDL-C level of <1.8 mmol/L; patients who had reached an LDL-C level of <1.4 mmol/L and achieved a ≥50% LDL-C reduction; and patients who had reached an LDL-C level of <1.4 mmol/L.^[7]^

Patients were divided into the following 2 groups: those patients who had been treated before the latest guideline release and those patients who had been treated after the release. To minimize selection bias from the real world, a propensity score-matched method was used. Patients treated before the guideline release were matched at a 1:1 ratio with randomly selected patients on the basis of nearest neighbor in terms of Mahalanobis distance, with a caliper of 0.05.^[8]^ The propensity score was estimated via a logistic regression model with the following variables: age, sex, index MI type, baseline LDL-C and past medical history, including hypertension, diabetes mellitus, heart failure, stroke, peripheral artery disease, chronic kidney disease, tobacco use, drinking history and premature family history of coronary artery disease.

Logistic regression was used to assess the association with intensive statin, ezetimibe and PCSK9 inhibitor usage together with treatment results between the 2 groups. The odds ratio (OR) and 95% confidence interval (CI) were reported. A 2-tailed *P* < .05 was considered to be statistically significant in all of the analyses. R software (http://www.R-project.org) was used for all of the statistical analyses.

## 3. Results

### 3.1. Patient characteristics

A total of 325 patients were enrolled in this study, including 141 patients who were admitted before the release of the latest guideline and 184 patients who were admitted after the release. The baseline characteristics are shown in Table 1. Patients who were admitted before the guideline release were quite similar to those patients admitted after the release on most of the characteristics, except for the fact that those patients admitted after the guideline release had less prior coronary heart disease and heart failure. The mean baseline LDL-C was 2.40 ± 0.89 mmol/L.

**Table 1:** Baseline characteristics before and after propensity score matching.

Variable	Unmatched				Propensity score matched			
	Overall	Before	After	*P* value	Overall	Before	After	*P* value
N	325	141	184		200	100	100	
Age, yr	64.09 (11.99)	63.72 (11.67)	64.38 (12.26)	.624	64.19 (12.04)	64.36 (11.79)	64.01 (12.34)	.838
Male sex	250 (76.92)	106 (75.18)	144 (78.26)	.602	154 (77.00)	75 (75.00)	79 (79.00)	.614
STEMI type	83 (25.54)	38 (26.95)	45 (24.46)	.702	57 (28.50)	27 (27.00)	30 (30.00)	.754
Prior medical history								
Hypertension	165 (50.77)	78 (55.32)	87 (47.28)	.185	100 (50.00)	51 (51.00)	49 (49.00)	.888
Diabetes	92 (28.31)	47 (33.33)	45 (24.46)	.102	52 (26.00)	25 (25.00)	27 (27.00)	.872
Dyslipidemia	144 (44.31)	70 (49.65)	74 (40.22)	.113	84 (42.00)	45 (45.00)	39 (39.00)	.474
CHD	163 (50.15)	88 (62.41)	75 (40.76)	<.001	94 (47.00)	48 (48.00)	46 (46.00)	.887
CABG	26 (8.00)	7 (4.96)	19 (10.33)	.119	12 (6.00)	6 (6.00)	6 (6.00)	>.999
Heart failure	27 (8.31)	20 (14.18)	7 (3.80)	.002	10 (5.00)	4 (4.00)	6 (6.00)	.746
Stroke	66 (20.31)	35 (24.82)	31 (16.85)	.103	36 (18.00)	15 (15.00)	21 (21.00)	.357
PAD	37 (11.38)	20 (14.18)	17 (9.24)	.224	22 (11.00)	12 (12.00)	10 (10.00)	.821
COPD	7 (2.15)	3 (2.13)	4 (2.17)	>.999	1 (0.50)	0 (0.00)	1 (1.00)	>.999
CKD	33 (10.15)	20 (14.18)	13 (7.07)	.055	16 (8.00)	8 (8.00)	8 (8.00)	>.999
Tobacco use	132 (40.62)	62 (43.97)	70 (38.04)	.335	78 (39.00)	41 (41.00)	37 (37.00)	.664
Drinking	104 (32.00)	48 (34.04)	56 (30.43)	.568	59 (29.50)	30 (30.00)	29 (29.00)	>.999
Premature CHD family history	27 (8.31)	14 (9.93)	13 (7.07)	.469	13 (6.50)	7 (7.00)	6 (6.00)	>.999
Vital signs								
HR, bpm	72.23 (13.22)	74.14 (15.25)	70.46 (10.80)	.038	71.06 (13.87)	72.24 (15.62)	69.77 (11.65)	.319
SBP, mm Hg	134.12 (18.33)	133.33 (18.58)	134.94 (18.12)	.532	134.75 (18.73)	133.68 (18.51)	136.12 (19.11)	.490
DBP, mm Hg	75.36 (11.66)	74.78 (11.92)	75.97 (11.42)	.469	74.53 (11.84)	73.60 (11.64)	75.72 (12.09)	.342
BMI, kg/m^2^	26.06 (3.36)	26.01 (3.32)	26.11 (3.41)	.847	26.26 (3.03)	26.05 (3.04)	26.50 (3.02)	.467
Baseline LDL-C, mmol/L	2.40 (0.89)	2.45 (0.85)	2.36 (0.91)	.333	2.45 (0.90)	2.50 (0.86)	2.40 (0.94)	.469

Data are expressed as mean ± SD or n (%).

BMI = body mass index, CABG = coronary artery bypass grafting, CHD = coronary heart disease, CKD = chronic kidney disease, COPD = chronic obstructive pulmonary disease, DBP = diastolic blood pressure, HR = heart rate, PAD = peripheral artery disease, SBP = systolic blood pressure, STEMI = ST-segment–elevation myocardial infarction.

After propensity score matching, post matching absolute standardized differences were <10% for all of the covariates. A total of 100 patients in each group were matched, and the characteristics of the 2 groups were recompared, which are also shown in Table 1. In the propensity score–matched population, there were no significant differences in any baseline characteristics (Table 1).

### 3.2. Initial lipid-lowering therapy and last LDL-C

Initial lipid-lowering therapy at discharge of index MI together with last LDL-C were compared between the 2 groups (Table 2). Overall, after a median follow-up time of 8.20 months (9.36 months in the group before guideline release and 7.31 months in the group after release), the mean LDL-C was 1.87 ± 0.59 mmol/L (1.87 ± 0.55 in the before group vs 1.88 ± 0.62 in the after group, *P* = .829). In addition, there was no significant difference in statin type between the 2 groups. The initial usage of intensive statin therapy was significantly decreased after guideline release (16.30% vs 29.08%, *P* = .018), whereas the usage of ezetimibe and PCSK9 inhibitors was significantly increased (16.30% vs 7.80%, *P* = .034; and 8.70% vs 2.13%, *P* = .024, respectively). Overall, the proportion of patients who reached an LDL-C level of <1.8 mmol/L was 52.62%, the proportion of patients who reached an LDL-C level of <1.4 mmol/L and achieved a ≥50% LDL-C reduction was only 4.31% and the proportion of patients who reached an LDL-C level of <1.4 mmol/L was 20.62%. Moreover, regardless of which criteria were used for the treatment target, there was no difference between the 2 groups.

**Table 2:** Initial lipid lowering therapy and treatment results between the 2 groups.

Variable	Unmatched				Propensity score matched			
	Overall	Before	After	*P* value	Overall	Before	After	*P* value
N	325	141	184		200	100	100	
Statins type, n (%)								
Pitavastatin	13 (4.30)	7 (5.22)	6 (3.57)	.836	8 (4.37)	6 (6.45)	2 (2.22)	.404
Pravastatin	4 (1.32)	2 (1.49)	2 (1.19)		1 (0.55)	1 (1.08)	0 (0.00)	
Rosuvastatin	59 (19.54)	29 (21.64)	30 (17.86)		31 (16.94)	15 (16.13)	16 (17.78)	
Simvastatin	2 (0.66)	1 (0.75)	1 (0.60)		1 (0.55)	1 (1.08)	0 (0.00)	
Atorvastatin	224 (74.17)	95 (70.90)	129 (76.79)		142 (77.60)	70 (75.27)	72 (80.00)	
Intensive statin therapy, n (%)	73 (22.46)	41 (29.08)	32 (17.39)	.018	45 (22.50)	28 (28.00)	17 (17.00)	.090
Ezetimibe, n (%)	41 (12.62)	11 (7.80)	30 (16.30)	.034	27 (13.50)	8 (8.00)	19 (19.00)	.039
PCSK9 inhibitor, n (%)	19 (5.85)	3 (2.13)	16 (8.70)	.024	13 (6.50)	3 (3.00)	10 (10.00)	.085
Last LDL-C, mmol/L, mean (SD)	1.87 (0.59)	1.87 (0.55)	1.88 (0.62)	.829	1.86 (0.59)	1.83 (0.55)	1.89 (0.63)	.443
Target 1, n (%)	171 (52.62)	74 (52.48)	97 (52.72)	>.999	106 (53.00)	55 (55.00)	51 (51.00)	.671
Target 2, n (%)	14 (4.31)	7 (4.96)	7 (3.80)	.814	10 (5.00)	6 (6.00)	4 (4.00)	.746
Target 3, n (%)	67 (20.62)	26 (18.44)	41 (22.28)	.477	40 (20.00)	19 (19.00)	21 (21.00)	.860

Target 1 indicates patients who had reached an LDL-C level of <1.8 mmol/L; Target 2, who had reached an LDL-C level of <1.4 mmol/L and achieved a ≥50% LDL-C reduction; Target 3, who had reached an LDL-C level of <1.4mmol/L.

After propensity score matching, the initial usage of intensive statin therapy was decreased after guideline release without statistical significance (17.00% vs 28.00%, *P* = .090), whereas the usage of ezetimibe and PCSK9 inhibitors was increased (19.00% vs 8.00%, *P* = .039; and 10.00% vs 3.00%, *P* = .085, respectively). Overall, the proportion of patients who reached an LDL-C level of <1.8 mmol/L was 53.00%, the proportion of patients who reached an LDL-C level of <1.4 mmol/L and achieved a ≥50% LDL-C reduction was only 5.00% and the proportion of patients who reached an LDL-C level of <1.4 mmol/L was 20.00%. Furthermore, regardless of which criteria were used for the treatment target, there was no difference between the 2 groups.

In the logistic regression models, the release of the guideline was associated with a statistically significantly increased use of ezetimibe (OR: 1.91; 95% CI: 1.21, 3.02; *P* = .005) after the multivariate analysis adjusted for possible confounders (Table 3). The usage of intensive statins was marginally decreased (OR: 0.68; 95% CI: 0.45, 1.03; *P* = .069), whereas PCSK9 inhibitors were marginally increased (OR: 1.31; 95% CI: 0.98, 1.76; *P* = .068).

**Table 3:** Initial lipid lowering therapy and proportion of achieved target associated with guideline release: unadjusted and multivariate adjusted analyses with propensity score matching.

Variable	Unadjusted			Multivariate adjusted*		
	OR	95% CI	*P* value	OR	95% C)	*P* value
Intensive statin therapy	0.60	0.34, 1.05	.073	0.68	0.45, 1.03	.069
Ezetimibe use	1.72	1.15, 2.57	.008	1.91	1.21, 3.02	.005
PCSK9 inhibitor use	1.98	0.96, 4.08	.064	1.31	0.98, 1.76	.068
Achieving Target 1	0.93	0.72, 1.20	.575	0.78	0.46, 1.32	.354
Achieving Target 2	0.77	0.32, 1.86	.563	0.96	0.59, 1.55	.856
Achieving Target 3	1.38	0.89, 2.13	.151	1.39	0.73, 2.66	.314

CI *=* confidence interval, OR *=* odds ratio.

* Adjusted for age, sex, MI type, and baseline LDL-C.

## 4. Discussion

In this real-world study, we found that the updated guidelines did lead to an increasing use of ezetimibe even when baseline lipid levels were not different before and after the release of the guidelines. The PCSK9 inhibitor also increased, but the increase was not statistically significant. As expected, the use of high-dose statins in AMI patients who were included after the guideline release also decreased, although no significant difference was found. In addition, there was no difference in the lipid level after the treatment between the 2 groups, and the number of patients who reached the LDL-C target remained small.

Increased LDL-C is an independent risk factor for atherosclerosis and remains the main lipid focus in clinical practice as the most extensively studied and targeted lipoprotein. In the last few decades, advances in genetics and analytical techniques, as well as a greater understanding of signaling molecules, have revealed multiple new targeting mechanisms for lipid-lowering therapies. Statins, ezetimibe or PCSK9 inhibitors by monoclonal antibodies have improved cardiovascular outcomes in randomized controlled trials, and the guidelines include their use.^[1]^ In recent years, studies on PCSK9 inhibitors have led to a better certainty of their cardiovascular benefits. The Effects of the PCSK9 Antibody Alirocumab on Coronary Atherosclerosis in Patients With Acute Myocardial Infarction (PACMAN-AMI) trial demonstrated that in patients with AMI, the addition of subcutaneous biweekly alirocumab to high-intensity statin therapy resulted in a significantly greater coronary plaque regression in noninfarct-related arteries after 52 weeks, compared to placebo treatment.^[9]^ As the longest follow-up data from a PCSK9 inhibitor study to date, the results of the Further cardiovascular Outcomes Research with PCSK9 Inhibition in subjects with Elevated Risk Open Label Extension (FOURIER-OLE) study demonstrated that long-term LDL-C lowering with evolocumab over a period of more than 8 years is safe and that long-term LDL-C lowering with evolocumab results in a greater reduction in cardiovascular events, including cardiovascular death, compared with delayed treatment initiation.^[10]^ Moreover, real-world studies have confirmed that PCSK9 inhibitors can improve lipid levels in a real-world setting in patients with high baseline LDL-C levels, despite receiving maximally tolerated LLTs, which is consistent with clinical trial results.^[11–13]^

In addition, other new lipid-lowering drugs are currently flourishing regarding their use.^[14]^ Inclisran is a small interfering RNA that targets PCSK9 mRNA to reduce PCSK9 synthesis, thus exerting lipid-lowering effects and producing a durable effect of 3–6 months when subcutaneously administered. The ORION 10 and ORION 11 trials enrolled patients with atherosclerotic cardiovascular disease (ASCVD) and patients with ASCVD or an ASCVD risk equivalent who had elevated LDL-C levels, despite receiving statin therapy at the maximum tolerated dose. The results showed that inclisran reduced LDL-C levels by 56% compared to placebo treatment.^[15]^ An ongoing randomized, double-blind, placebo-controlled trial (ORION-4, NCT03705234) is assessing the effects of inclisiran on clinical outcomes among people with ASCVD. Previous studies have shown that PCSK9 inhibitors have a high cost of benefit, and the addition of PCSK9 inhibitors to statin therapy is estimated to provide an additional quality-adjusted life year for a cost of $337,729.^[16]^ Inclisran and PCSK9 inhibitors have similar lipid lowering magnitudes, but inclisran has a longer duration of action than PCSK9 inhibitors; thus, inclisran may have better cost-effectiveness if it is effective in reducing cardiovascular events. Angiopoietin-like 3 (ANGPTL3) plays a prominent role in the regulation of lipid metabolism by inhibiting lipoprotein lipase and endothelial lipase. Thus, evinacumab, which is a fully human monoclonal antibody against ANGPTL3, has been developed. A phase 2 trial showed that evinacumab reduced LDL-C levels by more than 50% compared to placebo treatment in patients with refractory hypercholesterolemia.^[17]^ Bempedoic acid is an ATP-citrate lyase inhibitor, which is a cytosolic enzyme upstream of 3-hydroxy-3-methylglutaryl coenzyme A in the pathway for *de novo* cholesterol synthesis. Bempedoic acid is a prodrug that needs to be activated by the only enzyme present in hepatocytes that acts on the cholesterol biosynthesis pathway, thus avoiding muscle side effects compared to statins.^[18]^ Studies have shown that bempedoic acid reduces LDL-C levels by ~30% and by approximately 50% in combination with ezetimibe.^[19]^ Based on its lipid-lowering effects, bempedoic acid is the first nonstatin oral cholesterol-lowering drug approved by the Food and Drug Administration over the past 20 years. Subsequently, a meta-analysis showed that bempedoic acid in patients with hypercholesterolemia was associated with a lower risk of cardiovascular events and diabetes.^[20]^ An ongoing randomized, double-blind, placebo-controlled trial (CLEAR Outcomes, NCT02993406)^[21]^ is assessing the effects of bempedoic acid on the occurrence of major cardiovascular events in patients with or at a high risk for cardiovascular disease who are statin intolerant.

For ASCVD population lipid management guidelines, the LDL-C target value changed from 1.8 to 1.4 mmol/L; in addition, the latest guidelines recommend LDL-C being set as low as possible without setting a lower limit, and patients with AMI were recommended a target of LDL-C < 1.4 mmol/L and achieved a ≥50% reduction. There was a clear causal relationship between the increase in LDL-C value and ASCVD, and the minimizing of LDL particles and other APOB-containing lipoproteins was shown to reduce CV events.^[22]^ Although statins are highly effective in significantly reducing LDL-C at the initial dose, there is only a further reduction in LDL-C of approximately 5% to 6% after doubling the dose (which is known as the “rule of six”). The degree of LDL-C reduction is not fully parallel to cholesterol uptake because cholesterol uptake from the gastrointestinal tract is accelerated in response to a statin-induced decrease in serum.^[23]^ Ezetimibe is a relatively new cholesterol-lowering agent with a different mechanism from statins; thus, it provides another quality lipid-control treatment. It lowers cholesterol by inhibiting the absorption of LDL-C from the mucous membranes of the intestine.^[24]^ Previous studies have shown that intensive statin therapy increases the withdrawal rate and decreases patient compliance.^[25]^ Thus, the combination of ezetimibe and statins can improve patient compliance, which is beneficial for patients who cannot tolerate high-intensity statins; in addition, it does not reduce the lipid-lowering effect.^[26,27]^ Instead, the combination of the 2 drugs can play a synergistic role in reducing lipids.

The 2019 new guidelines indicate a higher grade of the combination use of statins and ezetimibe. However, the effect of the new guidelines on clinical practice is still unknown. With the new guidelines suggesting stricter requirements on lipid management, we hypothesize that patients’ total lipid standard-reaching rates would be higher (regardless of which criteria are used). However, our study did not obtain corresponding results. There are several possible reasons for this scenario. First, the new guidelines have not been popularized, and not all of the hospitals that were included in our study are teaching hospitals. In addition, the new target for lipid management and the indications for the use of new lipid-lowering drugs are not understood. Moreover, most of the previous studies on lipid-lowering therapy were followed up for at least 1 year, and the short follow-up time may be related to no significant difference in the last lipid level between the 2 groups. In addition, the increasing use of PCSK9 inhibitor was not statistically significant, which may be associated with the high price of the drug, the routine use of subcutaneous injections and the reimbursement of the drug in clinical practice.

Our study had several limitations. This study only included AMI patients in some hospitals in China; thus, it cannot represent the lipid treatment of all AMI patients in China. Only the initial lipid-lowering regimens were included in the study, and any changes in the therapeutic plan during the course of treatment were not counted. Moreover, this study did not include the incidence of cardiovascular events in patients after lipid-lowering therapy between the 2 groups. Therefore, we recommend that future studies consider the abovementioned factors. Expanding the study population, extending the follow-up time and increasing focus on the cost–benefit relationship of PCSK9 inhibitors are recommended.

There are 3 other things that need to be mentioned. First, a recent study of propensity score matching^[28]^ has shown that the upfront combination therapy of statin and ezetimibe is superior to statin monotherapy for all-cause death in patients with acute coronary syndromes, which suggests that in high-risk patients, such an approach, rather than a stepwise therapy approach, should be recommended. Second, the data regarding less frequent use of intensive statin regimen in our cohort are alarming. Statins have pleiotropic effects and only their high doses combined with ezetimib and PCSK-9 inhibitors are recommended.^[29,30]^ In fact, the number of patients on statin therapy didn’t decrease after this guideline but decrease the number of patients on high intensity statin; this finding is explained by the change of treatment management from statin monotherapy to statin plus ezetimibe +/- PCSK9 inhibitors or statin plus PCSK9 inhibitors or ezetimibe plus PCSK9 inhibitors. Third, circulating PCSK9 enhances platelet activation and that PCSK9 inhibitors reduce it, and one of the underlying mechanisms is that the treatment of PCSK9 inhibitors reduces platelet activation modulating soluble-NOX2-derived peptide activity and in turn oxidized-LDL formation in patients with heterozygous familial hypercholesterolemia.^[31]^

## 5. Conclusions

In this single-center real-world data analysis, after the release of the 2019 ESC/EAS guidelines, an increasing number of patients with a recent AMI were initially receiving ezetimibe and PCSK9 inhibitors. However, the proportion of patients who reached the guideline-recommended LDL-C target is still low in China. Given the current cost of PCSK9 inhibitors, the financial implications of the new guidelines may be substantial, and our findings highlight an urgent need for cost-effectiveness analyses of the 2019 ESC/EAS guideline recommendations.

## Author contributions

**Conceptualization:** Xiangqi Kong, Xiehui Chen.

**Data curation:** Gang He, Xiaoqing Quan, Zhixiong Tan, Fengjuan Yan.

**Formal analysis:** Xiehui Chen.

**Writing – original draft:** Xiangqi Kong, Gang He.
